# Biosynthetic Oligoclonal Antivenom (BOA) for Snakebite and Next-Generation Treatments for Snakebite Victims †

**DOI:** 10.3390/toxins10120534

**Published:** 2018-12-13

**Authors:** R. Manjunatha Kini, Sachdev S. Sidhu, Andreas Hougaard Laustsen

**Affiliations:** 1 Department of Biological Sciences, National University of Singapore, 14 Science Drive 4, Singapore 117543, Singapore; 2 Department of Molecular Genetics, The Donnelly Centre, University of Toronto, 160 College Street, Toronto, ON M5S 3E1, Canada; sachdev.sidhu@utoronto.ca; 3 Department of Biotechnology and Biomedicine, Technical University of Denmark, DK-2800 Kongens Lyngby, Denmark; ahola@bio.dtu.dk

**Keywords:** snakebite envenoming, neglected tropical diseases, antivenom, next-generation antivenom, recombinant antivenom, small molecule inhibitors

## Abstract

Snakebite envenoming is a neglected tropical disease that each year claims the lives of 80,000–140,000 victims worldwide. The only effective treatment against envenoming involves intravenous administration of antivenoms that comprise antibodies that have been isolated from the plasma of immunized animals, typically horses. The drawbacks of such conventional horse-derived antivenoms include their propensity for causing allergenic adverse reactions due to their heterologous and foreign nature, an inability to effectively neutralize toxins in distal tissue, a low content of toxin-neutralizing antibodies, and a complex manufacturing process that is dependent on husbandry and procurement of snake venoms. In recent years, an opportunity to develop a fundamentally novel type of antivenom has presented itself. By using modern antibody discovery strategies, such as phage display selection, and repurposing small molecule enzyme inhibitors, next-generation antivenoms that obviate the drawbacks of existing plasma-derived antivenoms could be developed. This article describes the conceptualization of a novel therapeutic development strategy for biosynthetic oligoclonal antivenom (BOA) for snakebites based on recombinantly expressed oligoclonal mixtures of human monoclonal antibodies, possibly combined with repurposed small molecule enzyme inhibitors.

## 1. Introduction

**Figure 1:**
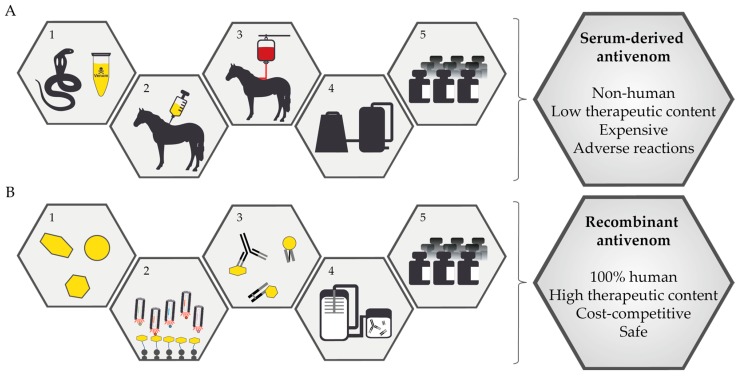
Schematic overview of the manufacturing processes for antivenoms. (**A**) Conventional plasma-derived antivenoms are manufactured through a five-step process. (**1**) Snakes are milked to obtain venom. (**2**) The venom is used to immunize a horse (or in some cases a sheep). (**3**) Upon completion of the immunization process, blood is drawn from the horse. (**4**) Plasma and erythrocytes are separated, and different precipitation techniques are used to isolate IgG antibodies from the plasma. (**5**) Following concentration and formulation, the antivenom is bottled and ready for use. (**B**) In contrast, recombinant antivenoms based on monoclonal antibodies and/or antibody fragments can be developed through a very different, and much more defined, five-step process. (**1**) Different techniques are used to identify medically important venom toxins (e.g., toxicovenomics). (**2**) Using phage display selection (or other antibody discovery techniques), monoclonal antibodies are discovered against the medically relevant toxins. (**3**) Different formats of monoclonal antibodies may be combined to formulate an oligoclonal mixture of monoclonal antibodies that each target different key toxins. (**4**) The oligoclonal antibody mixture is manufactured using cell cultivation techniques, such as single-batch expression technologies. (**5**) Upon purification and formulation, the recombinant antivenom is bottled and ready for use. Drawbacks of conventional plasma-derived antivenoms and the corresponding benefits of recombinant antivenoms are presented in the right side of the figure.

Snakebite is a serious menace in tropical countries and was recognized as a “neglected tropical disease” by the World Health Organization in 2017 [1]. Every year, more than 1.8–2.7 million cases of snakebite envenoming in human victims occur, resulting in 80,000–140,000 deaths and at least twice as many disabling morbidities around the world [2]. Most of the victims are in their productive age (between 20–40 years) and are often main breadwinners, leading to a great negative impact on the economics of their families. India has the highest number of deaths in the world due to snakebites (more than 46,000 [3]), predominantly caused by the “big four” snakes: Indian cobra (*Naja naja*), Common krait (*Bungarus caeruleus*), Russell’s viper (*Daboia russelii*), and Saw-scaled viper (*Echis carinatus*) [4]. In October 2018, several like-minded basic scientists and clinicians came together at the Live and Let Live: Snakebite Cure Symposium at the Nextgen Genomics, Biology, Bioinformatics, and Technologies Conference in Jaipur, India to find a sustainable solution to the Indian snakebite envenoming challenge. Scientific discussions at this event concluded in agreement that the concept presented in this article is likely to be a promising avenue to follow for the development of next-generation antivenom with improved therapeutic properties. In this present concept, we propose the use of recombinant human antibodies and small molecule inhibitors to eventually replace horse-derived antivenoms (Figure 1). These next-generation treatments will have better efficacy and a reduced level of adverse reactions compared to current therapies.

## 2. Current Treatment for Snakebite Victims

Snakebite envenoming is a severe medical emergency that can cause multiple organ failure. Thus, it requires quick and timely treatment of the victims. Currently, the only accepted treatment for snakebite envenomings involves intravenous administration of conventional antivenoms, which comprise antibodies or antibody fragments derived from the plasma of larger mammals (typically horses) that have been immunized with snake venom(s) (Figure 1A) [5,6]. Unfortunately, the use of such heterologous antivenoms has numerous inherent drawbacks:

**1.**  **Inability to abrogate local tissue damage:** Snakebites from several snake species cause severe local tissue damage, leading to disfigurement, amputation, and permanent disability. The administration of antivenoms in most cases fails to neutralize this catastrophic pathology, as the heterologous antibodies or antibody fragments in antivenoms have insufficient pharmacokinetics to reach and neutralize toxins in deep tissue before these have started exerting their toxic functions [2].
**2.**  **Allergic reactions and anaphylactic shock:** The administration of antivenoms, which are foreign horse-derived antibodies, may lead to acute anaphylactic shock in snakebite victims, which has been demonstrated to be the case for >40% for certain antivenoms [7,8,9,10]. These life-threatening adverse reactions must be managed by attending clinicians.
**3.**  **Serum sickness:** Serum sickness is a delayed response to antivenom administration that occurs for 5–56% of treated victims for certain antivenoms [11,12,13]. The incidence of serum sickness is poorly defined, mostly because patients rarely return to health centers or they are not adequately followed after hospital discharge. Despite best efforts, typical antivenoms contain only 5–36% snake venom toxin-binding antibodies [14,15,16]. The ability of these antibodies to neutralize snakebite pathologies depends on the proportion of toxin-neutralizing antibodies and their pharmacokinetics. Hence, a significant number of antivenom vials are administered to each snakebite victim, with extreme cases requiring as much as 15 g of heterologous antibody protein [17]. Such a high dose administration increases the probability of serum sickness.
**4.**  **Inability to neutralize snake venoms from different regions:** Snake venoms exhibit significant geographic variations in their toxin composition [18,19,20,21,22,23,24,25,26,27,28,29]. These variations are due to local adaptation, differences in diet, and ontogeny [30]. In a large country, like India, it would be ideal to pool venoms from various regions when designing immunization mixtures to overcome this drawback.
**5.**  **Complex manufacturing processes:** Antivenom manufacture is complicated by the dependence of polyclonal antibodies on two biological systems, namely representative snake venoms and individual horse immune systems.

Some of the above drawbacks make clinicians recalcitrant to treat snakebite victims with antivenom. These drawbacks—taken together with poor transportation to quickly reach primary health centers, unavailability of antivenoms, and poor training of clinicians—lead to a large number of snakebite-induced deaths.

## 3. Next-Generation Snakebite Therapy

Based on the above limitations, we opined that there is an unmet medical need for significantly improved treatments for snakebite victims. In this regard, we considered the use of recombinant antivenoms containing oligoclonal mixtures of human monoclonal antibodies along with small molecule inhibitors.

### 3.1. Biosynthetic Oligoclonal Antibodies (BOA) for Snakebite

In the last couple of decades, human therapeutic antibodies have become the mainstay in the development of biologics for the treatment of various human diseases [31]. Human antibodies and their fragments are useful in acute and chronic treatments. Such applications have led to improved technologies for the production of high-quality human antibodies in large amounts. High quality, efficacious biosynthetic oligoclonal antibodies (BOA) for snakebite requires a cocktail of human antibodies that target most or all of the key toxins that are responsible for snakebite-induced pathophysiology (Figure 1B) [32,33,34]. The key toxins to be targeted could be elucidated using toxicovenomic approaches, including the assessment of potential toxin synergism [35,36,37,38]. BOAs may contain 20–40 or more toxin-neutralizing antibodies for a given number of particular snake venoms, but critically and in contrast to horse-derived polysera, these BOAs would be precisely defined mixtures of carefully selected recombinant human antibodies [39]. Additionally, BOAs can be designed to be monovalent or polyvalent against different snake species, depending on the number and specificity of monoclonal antibodies included in the final formulations.

To achieve as facile and low-cost development as possible, it is relevant to piggyback on existing technologies for selection, production, and characterization of therapeutic antibodies [40]. One of the technologies that has been identified as particularly promising for developing therapeutic antibodies against snake venom toxins is phage display [41,42]. This technology allows for the development of therapeutic monoclonal antibodies that are fully human to avoid allergenic adverse reactions and loss of efficacy in human recipients. Antibody phage display technology allows for the discovery and maturation of fully human antibodies in vitro [43]. In essence, phage display technology simulates the human immune system in the lab, by employing libraries of antibody-displaying bacteriophages [41,44,45,46] to select for human antibody fragments in vitro [47]. The phage display approach to antivenom research circumvents meticulous immunization and screening protocols and overcomes the high toxicity and low immunogenicity of the target protein. Following conversion from the typically employed single-chain variable fragment (scFv) or antigen-binding fragment (Fab) format to the full human immunoglobulin G (IgG) format, these antibodies can be expressed in mammalian cells to ensure correct folding and post-translational modifications [48]. Following this approach, Laustsen et al. have recently reported the development of the first experimental BOAs consisting of in vivo neutralizing human IgG antibodies against toxins from the black mamba [32]. Additionally, manufacturing oligoclonal mixtures of IgGs has been demonstrated to be cost-competitive [39,49].

However, several hurdles in the development of next-generation antivenoms were identified: (a) There is a lack of point-of-care diagnostic kits for snakebites that are simple, reliable, and fast. Preferably, such kits would be both qualitative and quantitative. Such kits will be essential for patient stratification during clinical trials with BOAs to ensure that patients are treated with a BOA that is efficacious against envenomings by the perpetrating snake species in each clinical trial case; (b) there is a paucity of information on key target toxins in each venom that have to be neutralized to achieve better efficacy in terms of reduction in mortality and morbidity; (c) a streamlined pathway has yet to be defined to enable evaluation of the efficacy and safety of BOAs to enable approval and marketing; and (d) the specific formulation of a given (monovalent or polyvalent) BOA needs to be defined in terms of which snake species it should be efficacious against, so that relevant monoclonal antibodies that target all the key medically toxins present in the venoms of these species are included.

BOA will have many potential advantages over conventional horse-derived antivenoms:

**1.**  **Compatibility with human victims:** BOA will contain only human antibodies and will thus be compatible with treatment of human patients [50].
**2.**  **Enriched for toxin-neutralizing antibodies:** Horse-derived antivenoms contain both toxin-neutralizing and toxin-binding antibodies, but only toxin-neutralizing antibodies are useful for abrogating the pathophysiology of envenomation [33]. Antibody production in animals occurs due to the natural immune response, and there is no control over the antibody clones that expand and produce antibodies [51]. Therefore, horse antibodies show significant differences in their neutralizing capacities. In addition, these antivenoms may also contain antibodies raised against irrelevant infections, to which horses used for antivenom manufacture may have been exposed. Consequently, horse-derived antivenoms contain a small percentage of toxin-neutralizing antibodies [5]. In contrast, recombinant antibodies can be selected precisely for toxin-neutralizing ability. Therefore, the BOA will be enriched for toxin-neutralizing antibodies.
**3.**  **Consistent and reproducible production:** The production of polyclonal antibodies in horses is highly variable, and there will always be inherent batch-to-batch variations. The quality of BOAs will provide excellent consistency and reproducibility, and thus, batch-to-batch variation will be obviated [39,50].
**4.**  **Tailor-made antibodies with optimal pharmacokinetics (PK) and pharmacodynamics (PD):** Different toxins in snake venoms exhibit distinct biodistribution, PK, and PD, which often contributes to multi-organ failure in snakebite victims. Neutralization of such varied properties of toxins requires antibodies with appropriate biodistribution, PK, and PD. This can be achieved by utilizing full-length immunoglobulin G (IgG) antibodies, antibody fragments, or alternative non-antibody-based binding proteins [35,50]. In the preparation of BOAs, it is possible to include a mixture of full-length antibodies and/or fragments based on the properties of each toxin (toxicokinetics). Tailor-made mixtures of antibodies cannot be produced from horse-derived polyclonal antibodies, but are possible in BOA technology.
**5.**  **Better safety profile:** Highly compatible, toxin-neutralizing antibodies with suitable PK and PD are expected to have better safety profiles compared to horse-derived antivenoms [50]. Thus, intravenous administration of a BOA should not cause acute (allergic and anaphylactic) or delayed (serum sickness) reactions.
**6.**  **Rapid administration of antivenoms:** Because of inherent acute allergic and anaphylactic reactions, horse-derived antivenoms are administered to snakebite victims only after the victim develops symptoms and has reached a hospital setting. Such delays lead to poor treatment outcomes. The better safety profile of the BOA will allow quicker administration, for example, during transportation to the hospital, thus likely allowing for improved treatment outcomes.
**7.**  **Acceptance among clinicians:** Poor efficacy compounded with acute (allergic and anaphylactic) and delayed (serum sickness) reactions has kept many clinicians from venturing to treat snakebite victims with antivenom. With better efficacy and safety profiles, BOA will help in the acceptance of treatment of snakebite victims.
**8.**  **Geographic variation of venoms:** Most of the geographic variation in venom composition is due to differences in the abundance of specific toxins in venoms from snake specimens obtained from different regions. Such variations, at times, will make horse-derived antivenoms raised against venoms from one region ineffective against venoms from the same species in another region. Additionally, venoms from the same snake species from different regions may have one or more distinct/unique toxins. In both these cases, the problems can be overcome with BOA by simply including more antibodies or additional antibodies against all offending toxin(s). Such additions will not affect the safety profile of the BOA due to the compatibility of human monoclonal antibodies with the human immune system.
**9.**  **Cross-reactivity with other snake venom toxins:** Some toxin-neutralizing antibodies neutralize related toxins not only from the same species, but also from different species [52]. If such cross-reactivity is intelligently engineered into the monoclonal antibodies during development, it may help in preparing polyvalent BOAs from a stock of a limited number of human antibodies [53].
**10.**  **Elimination of interaction with live snakes:** The production of BOA does not require a continuous supply of snake venoms. This will eliminate the need for collecting venoms from wild or captive snakes, thus reducing accidental bites [5,50].
**11.**  **Abrogation of local tissue damage:** In most cases, horse-derived antivenoms fail to abrogate local tissue damage induced by snake venom toxins [2]. This inability could be either due to a lack of antibodies that neutralize the offending toxin(s) or to the toxin(s) initiating local tissue damaging processes before being neutralized by antibodies [54]. It may be possible to find suitable human antibodies that could neutralize offending toxins. Alternatively, enzymatic processes leading to local tissue damage could be neutralized using small molecule enzyme inhibitors [55,56] (see below). Finally, by having an improved safety profile, it might be possible to administer BOA during transportation en route to the hospital, thereby minimizing the time that the locally-acting snake toxins can exert their toxic actions around the bite wound.
**12.**  **Potential prophylactic use of BOA:** The better safety profile of BOA could be of prophylactic use for people who will be exposed to snakebite hazards. The longer PK of full-length antibodies (IgGs), which typically have half-lives of several weeks [50], could provide excellent prophylactic protection, which could reduce mortality, morbidity, and intensity of pathophysiological impact of snakebite.

### 3.2. Small Molecule Enzyme Inhibitors

**Figure 2:**
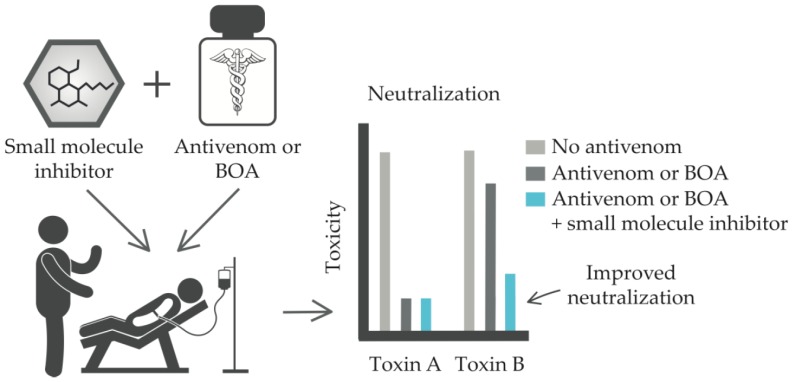
Schematic representation of how small molecule inhibitors may be used in combination with conventional antivenom or biosynthetic oligoclonal antivenom (BOA) against snakebite to strategically neutralize key toxins that are poorly neutralized by the antivenom or BOA.

Snake venoms contain enzymes, including phospholipases A_2_, metalloproteases, serine proteases, L-amino acid oxidases, nucleotidases, and hyaluronidases [2,5,57,58,59]. In addition to the digestion of prey, these venom enzymes contribute to various pharmacological functions. They may also contribute to various pathophysiologies of envenomation, including local tissue destruction and damage. Over the last several decades, a number of natural and synthetic molecules have been evaluated for their ability to inhibit various enzymes found in snake venoms, and some of these molecules have been tested in human patients for treatment of other diseases [5,55,60,61]. Such inhibitors could be repurposed for the treatment of snakebite victims, potentially in combination with current antivenoms or next-generation antivenoms (Figure 2) [60,62].

The key hurdle in the use of such inhibitors is evaluating their efficacy in inhibiting offending snake venom enzymes, both in vitro and in vivo (including in treatment mode). These molecules should also be evaluated for their in vivo efficacy in combination with horse-derived antivenoms as well as BOA. Small molecule enzyme inhibitors could provide several potential advantages.

**1.**  **Increased treatment window:** Treatment for envenomation should ideally start within a short time period following snakebite, as mortality and morbidity increase significantly beyond this window. Small molecule enzyme inhibitors may substantially increase this time window, if they can be administered in the field setting (i.e., if they are stable at elevated temperatures and orally available), and could thus provide more time to reach hospital care.
**2.**  **Fast and effective tissue penetration:** The small size of the inhibitors may allow rapid distribution to all ‘compartments’ in vivo, if such inhibitors exhibit fast diffusion kinetics and effective tissue penetration [5,54,61].
**3.**  **Validated safety in humans:** As many of these small molecule inhibitors have been evaluated for their toxicity in human recipients, they have already been proven sufficiently safe for use in the treatment of snakebite envenoming.

## 4. Conclusions

Mortality and morbidity due to snakebite envenoming represents a solvable international disaster. For more than 100 years, animal-derived antivenoms have been the most accepted treatment for snakebite victims (Figure 1A). Although they significantly reduce mortality and morbidity, these antiquated antivenoms are beset with inherent limitations, including a high propensity to cause adverse reactions, complex manufacturing processes, low content of toxin-neutralizing antibodies, and an inability to effectively neutralize locally-acting toxins in distal tissues. The concept of using biosynthetic oligoclonal antivenom (BOA) for snakebites proposes to use modern well-established technology to overcome the limitations of existing treatments through using oligoclonal mixtures of human monoclonal antibodies and repurposed enzyme inhibitors (Figure 1B). Being based on human therapeutic antibodies, a BOA would not cause allergenic reactions in human recipients. Also, since the antibodies included in a BOA would be specifically selected as toxin-neutralizing recombinant monoclonal antibodies (both for monovalent and polyvalent BOAs), a BOA could be manufactured to only comprise therapeutically active antibodies by industrially standardized manufacturing processes for oligoclonal antibody mixtures. Finally, the combined use of small molecule inhibitors together with a BOA could lead to improved neutralization of toxins in distal tissue by improving pharmacokinetics (Figure 2).
